# Predicting intra-abdominal candidiasis in elderly septic patients using machine learning based on lymphocyte subtyping: a prospective cohort study

**DOI:** 10.3389/fphar.2024.1486346

**Published:** 2024-12-12

**Authors:** Jiahui Zhang, Guoyu Zhao, Xianli Lei, Na Cui

**Affiliations:** Department of Critical Care Medicine, State Key Laboratory of Complex Severe and Rare Diseases, Peking Union Medical College Hospital, Chinese Academy of Medical Science and Peking Union Medical College, Beijing, China

**Keywords:** intra-abdominal candidiasis, elderly, sepsis, lymphocyte subtyping, risk stratification, machine learning, nomogram

## Abstract

**Objective:**

Intra-abdominal candidiasis (IAC) is difficult to predict in elderly septic patients with intra-abdominal infection (IAI). This study aimed to develop and validate a nomogram based on lymphocyte subtyping and clinical factors for the early and rapid prediction of IAC in elderly septic patients.

**Methods:**

A prospective cohort study of 284 consecutive elderly patients diagnosed with sepsis and IAI was performed. We assessed the clinical characteristics and parameters of lymphocyte subtyping at the onset of IAI. A machine-learning random forest model was used to select important variables, and multivariate logistic regression was used to analyze the factors influencing IAC. A nomogram model was constructed, and the discrimination, calibration, and clinical effectiveness of the model were verified.

**Results:**

According to the results of the random forest and multivariate analyses, gastrointestinal perforation, renal replacement therapy (RRT), T-cell count, CD28+CD8+ T-cell count and CD38+CD8+ T-cell count were independent predictors of IAC. Using the above parameters to establish a nomogram, the area under the curve (AUC) values of the nomogram in the training and testing cohorts were 0.840 (95% CI 0.778-0.902) and 0.783 (95% CI 0.682-0.883), respectively. The AUC in the training cohort was greater than the *Candida* score [0.840 (95% CI 0.778-0.902) vs. 0.539 (95% CI 0.464-0.615), p< 0.001]. The calibration curve showed good predictive values and observed values of the nomogram; the DCA results showed that the nomogram had high clinical value.

**Conclusion:**

We established a nomogram based on the T-cell count, CD28+CD8+ T-cell count, CD38+CD8+ T-cell count and clinical risk factors that can help clinical physicians quickly rule out IAC or identify elderly patients at greater risk for IAC at the onset of infection.

**Clinical Trial Registration:**

[chictr.org.cn], identifier [ChiCTR2300069020].

## 1 Introduction

Elderly individuals exhibit numerous distinct physical characteristics compared to their younger counterparts. Ageing is intricately linked to numerous alterations in organ functionality, encompassing an augmentation in frailty, a decrement in activities of daily living, a reduction in mobility, and cognitive decline (Vincent and Creteur, 2022). A significant disadvantage among the elderly population is the diminished barriers to pathogens, resulting in a heightened susceptibility to infection. The incidence of sepsis increases with advanced age. Furthermore, aging results in diverse physiological changes within the immune system, which are collectively referred to as immunosenescence, thereby increasing the susceptibility of elderly patients to fungal colonization and infections (Akinosoglou et al., 2023). Consequently, elderly patients constitute a distinct subgroup within the septic patient population, necessitating tailored management strategies.

Intra-abdominal candidiasis (IAC) poses a significant threat to life, with estimates primarily stemming from immunocompromised individuals. Despite its relatively low cumulative incidence of 1.84 cases per 1,000 intensive care unit (ICU) admissions (Bassetti et al., 2019), IAC is associated with a significant mortality rate of up to 60%, highlighting its severity and importance in critical care settings (Vergidis et al., 2016). However, there is a paucity of data specifically focusing on the elderly population, who are potential immunodeficient hosts with age-related immune dysregulation. Except for the established risk factors, the high rate of comorbidity, malnutrition, and polypharmacy, which are common in the elderly, could intervene the clinical management of IAC. The incidence and mortality of IAC increases with advanced age (Bassetti et al., 2015). However, the risk factors and techniques guiding the rapid recognition for IAC in elderly septic patients are lacking.

The diagnosis of IAC poses a significant challenge in elderly septic patients with intra-abdominal infection (IAI), often leading to excessive or delayed utilization of antifungal agents. Accurate diagnosis of IAC necessitates the isolation of *Candida* species via traditional mycological culture techniques, albeit with the potential for prolonged turnaround times. In consideration of the detrimental outcomes stemming from delayed treatment, antifungal agents are frequently administered prior to obtaining culture results, taking into account clinical risk factors and the comprehensive context of the patient’s condition. While research is ongoing for certain biomarkers and clinical risk factors, such as (1,3)-β-D-glucan (BDG), the currently available clinical scoring systems are insufficient in identifying patients with a high risk of IAC (Leroy et al., 2016; Bailly et al., 2017). *Candida* spp. can affect immune status by decreasing immunorecognition or overactivating the inflammatory response (Romani, 2011). T cells constitute a crucial component of the adaptive immune system, and play a pivotal role in generating immunological memory against invading fungi. Previous investigations conducted by our research team have demonstrated a strong association between CD8+ T-cell impairment and the development of invasive candidiasis (IC) among ICU patients. This finding underscores the potential utility of lymphocyte subtyping as a rapid diagnostic tool with significant clinical implications for the early identification of IAC (Zhang et al., 2019; Zhang et al., 2022).

Based on our current understanding, prior investigations into IAC have exhibited limitations in scope and comprehensiveness, often focusing narrowly on clinical manifestations (Bassetti et al., 2022; Novy et al., 2023). Furthermore, the extant literature has not yet arrived at a definitive consensus regarding the effectiveness of lymphocyte subtyping in identifying IAC, and at present, there exist no established models capable of swiftly stratifying elderly patients into distinct high- and low-risk categories. With the objective of enhancing the diagnostic precision of IAC and enabling the prompt commencement of antifungal therapy, this study aimed to identify elderly septic patients with an elevated risk of developing IAC. Additionally, we assessed the potential value of lymphocyte subtyping in the early prediction of IAC and formulated a nomogram that integrates lymphocyte subtyping and clinical parameters for the rapid diagnosis of IAC.

## 2 Methods

### 2.1 Study design and participants

**FIGURE 1:**
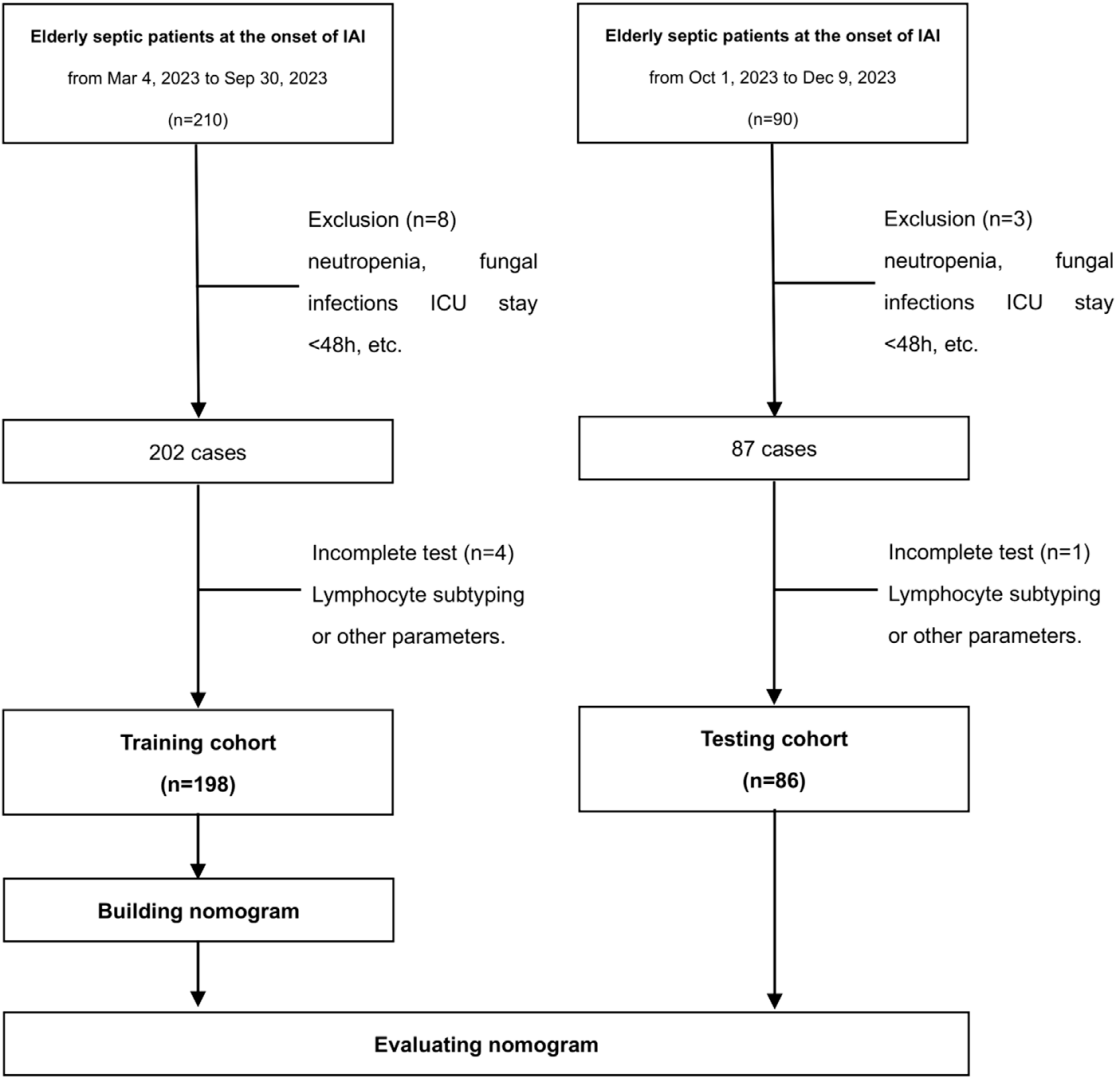
Flowchart of the study population.

This prospective, single-center, noninterventional cohort study was conducted at Peking Union Medical College Hospital (PUMCH). Elderly septic patients with IAI were enrolled consecutively (Figure 1). The training cohort was recruited from 4 March 2023 to 31 December 2023. The testing cohort comprised patients enrolled from the same center, spanning from 1 January 2024 to 30 June 2024. Strict adherence to the inclusion criteria ensured that only eligible patients were recruited for the study. Prior to their inclusion, written informed consent was obtained from either the patients themselves or their legal guardians. The Ethics Commission of PUMCH granted ethical approval for the conduct of this study (approval number: I-22PJ998). The inclusion criteria were: (i) age≥65 years, (ii) expected duration of ICU stay exceeding 48 h, (iii) sepsis diagnosis, and (iv) IAI diagnosis. The diagnoses of sepsis and IAC were made by experienced clinicians. Sepsis was defined in accordance with the official criteria outlined in the Sepsis 3.0 definition (Singer et al., 2016). An episode of IAC was defined as follows: (i) the detection of *Candida* by direct microscopic examination or growth in culture from purulent or necrotic intra-abdominal specimens obtained during surgery or by percutaneous aspiration; (ii) the detection of *Candida* growth from bile, intrabiliary ducts devices, and biopsy of intra-abdominal organs; (iii) the detection of *Candida* growth obtained from patients with secondary and tertiary peritonitis in the absence of any other pathogen; or (iv) the detection of *Candida* growth from drainage tubes only if placed less than 24 h before the cultures (Bassetti et al., 2015). The exclusion criteria were: (i) pregnancy or lactation, (ii) neutropenia defined as an absolute neutrophil count less than 500/mm^3^ at baseline, (iii) other fungal infections, excluding those caused by *Candida* species, (iv) a diagnosis of invasive candidiasis excluding IAC, or (v) death within a 48-h period.

### 2.2 Data collection

The clinical data included baseline demographics, comorbidities, and the identification of recognized risk factors for IAC. The identified clinical risk factors for IAC were broad-spectrum antibiotic use, high-dose corticosteroid use, an Acute Physiology and Chronic Health Evaluation (APACHE) II score of 15 or higher, need for renal replacement therapy (RRT), the status of BDG positivity, emergency gastrointestinal/hepatobiliary (GIT/HPB) surgery, total parenteral nutrition, and gastrointestinal perforation (Bassetti et al., 2015; Pappas et al., 2016; Bassetti et al., 2022; Novy et al., 2023). The duration of corticosteroid use was at least 3 weeks (De Pauw et al., 2008). The definition of previous *Candida* colonization was the isolation of *Candida* species from cultures obtained from at least two of the following sources: urine, skin, stool, wound sites, respiratory tract secretions, and drains that had been in place for 24 h or less (Bassetti et al., 2021). Peripheral blood samples were obtained from each patient upon the onset of IAI for the purpose of detecting lymphocyte subsets and other pertinent parameters. Subsequently, lymphocyte subsets and immunological parameters were promptly analyzed and determined by the laboratories affiliated with PUMCH using peripheral blood samples. Peripheral blood mononuclear cells (PBMCs) were isolated and stained with combinations of different fluorescent monoclonal antibodies and subsequently analyzed by flow cytometry using a three-color EPICS-XL flow cytometer from Beckman Coulter (Brea, CA, United States). CD3+ cells were gated to identify T cells, CD4+CD3+ cells were gated to identify CD4+ T cells, and CD8+CD3+ cells were gated to identify CD8+ T cells. An independent clinical research organization conducted monitoring to guarantee the adherence to good clinical practices in accordance with the Chinese government’s regulations.

### 2.3 Statistical analysis

For the two-class prediction model, one of the sample size calculation methods proposed in the article (Riley et al., 2020) is $n=\exp\;\left(\frac{-0.508+0.259\ln\varphi +0.504\ln P-\ln\left(MAPE\right)}{0.544}\right),$ where φ is the proportion of ending events (φ = 0.33), P is the number of predictors (*p* = 5), MAPE is the average absolute error between the observed and true outcome probability (MAPE = 0.05). According to the above formula, the sample size of the study is calculated as at least 255, less than the sample size 284 included in this study.

Variables that follow a normal distribution are typically summarized using the mean and standard deviation (SD). Significant differences among these variables were determined through the application of either Student’s t-test or one-way ANOVA. Nonnormally distributed variables are summarized using the median and interquartile range (IQR), and significant differences were determined using the Mann-Whitney U test or Kruskal–Wallis test. Categorical variables are represented as proportions and analyzed statistically through the chi-squared test or Fisher’s exact test, depending on the circumstances. Parameters with missing data of more than 20% were excluded from the final dataset. Parameters with missing data of less than 20% were interpolated using the missForest package. MissForest package is a non-parametric method that utilizes random forests to impute missing values, suitable for both continuous and categorical variables. Its core algorithm is to use known variables as independent variables to establish a random forest to predict missing values. It yields an out-of-bag (OOB) imputation error estimate. We conducted a thorough assessment of the collinearity among the variables, utilizing both the variance inflation factor (VIF) and correlation statistics as our guiding metrics. A VIF of 5 or more was used to identify multicollinearity. The selection processes for variables were enhanced by the integration of machine learning techniques. Random forest variable selection was performed with the “randomForest” package. The random forest model assigned a numerical value, indicating the importance of each variable, based on its relevance to the overall model. In our study, we built a random forest model to screen out risk factors to predict intra-abdominal candidiasis (IAC) in elderly septic patients using the “randomForest” package of R. The parameters were set as follow: ntrees = “500” and mtry = “3”. The *X*-axis represents the number of trees and *Y*-axis represents the error value of 10-fold cross validation. In this way, we selected the tree with a minimum error value as the optimal model for predicting the incidence of IAC. A random forest method was chosen because it is non-parametric and builds upon the positive attributes of the popular decision tree method such as providing implicit feature selection, and decreased sensitivity to outliers compared to other classification techniques such as logistic or linear regression. Given the novel nature of this classification model, minimal *a priori* feature was preferred. Furthermore, by combining the results of multiple individual decision trees, it follows that a combination of all resultant outputs may result in a higher predictive accuracy than each constituent tree alone, especially with complex and high-dimensional data. The combination of this majority voting approach on sub-samples of the data is known as boot-strap aggregating. Bagging decreases the likelihood of overfitting and improves model generalization by decreasing outlier influence and model variance. This then provides a unique advantage when encountering high-dimensional data with complex interactions (Oliva et al., 2022). The “glm” package was utilized for univariate and multivariate logistic regression analyses. The calibration plots and DCA were generated using the “rms” and “rmda” packages, respectively. A nomogram was constructed using the “rms” package. A statistical analysis was conducted to determine significant differences in the area under the curve (AUC) between the two receiver operating characteristic (ROC) curves, employing the Delong test for comparison. Discrimination of the models was assessed using the net reclassification index (NRI) and the integrated discrimination index (IDI). We use a fivefold cross-validation technique to address concerns of model overfitting. Statistical analysis was conducted using R (version 4.2.1) and DCPM (version 3.37, Jingding Medical Technology Co., Ltd.). The results with p values less than 0.05 were considered significant.

## 3 Results

### 3.1 Patient characteristics

There were no significant differences in any of the clinical features between patients in the training and testing cohorts (Table 1). However, significant differences were detected between IAC patients and non-IAC patients in the training cohort, including immune system disease, BDG positivity, gastrointestinal perforation, CD28+CD8+ T cell-count, DR+CD8+ T cell-count and CD38+CD8+ T cell-count (p< 0.05, Table 2).

**TABLE 1:** Baseline characteristics of enrolled patients in the training and testing cohorts.

Variables	Traing cohort (n = 198)	Testing cohort (n = 86)	p-value
Mean age (y)	73 [69; 80]	75 [70; 81]	0.439
Gender, male, n (%)	125 (63.1)	56 (65.1)	0.853
Diabetic mellitus, n (%)	36 (18.2)	17 (19.8)	0.881
Chronic renal failure, n (%)	31 (15.7)	12 (14.0)	0.851
Hepatic falure, n (%)	6 (3.0)	0 (0.0)	0.183
Solid tumor, n (%)	64 (32.3)	22 (25.6)	0.319
Immune system disease, n (%)	6 (3.0)	2 (2.3)	0.999
Hematological disease, n (%)	9 (4.6)	2 (2.3)	0.513
APACHE II score≥15	146 (73.7)	64 (74.4)	0.999
SOFA score	8 [5; 10]	8 [5; 11]	0.970
BDG positive, n (%)	36 (18.2)	22 (25.6)	0.207
Need for mechanical ventilation, n (%)	192 (97.0)	84 (97.7)	0.999
Need for vasopressor, n (%)	185 (93.4)	79 (91.9)	0.823
Need for RRT, n (%)	45 (22.7)	22 (25.6)	0.713
High-dose corticosteroids receipt, n (%)	37 (18.7)	13 (15.1)	0.578
Broad-spectrum antibiotics receipt, n (%)	167 (83.3)	68 (79.1)	0.363
Total parenteral nutrition, n (%)	174 (87.9)	77 (89.5)	0.843
Emergency GIT/HPB surgery, n (%)	133 (67.2)	52 (60.5)	0.340
Gastrointestinal perforation, n (%)	92 (46.5)	42 (48.8)	0.811
WBC (cells/mm^3^)	10,710 [8,530; 14,640]	11,570 [8,120; 16,190]	0.458
LY (cells/mm^3^)	622 [449; 958]	604.5 [408; 879]	0.452
T cells (cells/mm^3^)	450 [267; 686]	427 [286; 638]	0.261
CD4+T (cells/mm^3^)	258 [147; 413]	250 [149; 418]	0.743
CD28+CD4+T (cells/mm^3^)	230 [134; 370]	230 [129; 345]	0.694
CD8+T (cells/mm^3^)	130 [84; 209]	129 [68; 205]	0.823
CD28+CD8+T (cells/mm^3^)	48 [26; 77]	45 [28; 75]	0.754
DR+CD8+T (cells/mm^3^)	61 [35; 108]	67 [32; 133]	0.493
CD38+CD8+T (cells/mm^3^)	65 [40; 115]	70 [33; 132]	0.651
T4/T8	2.02 [1.23; 2.91]	2.06 [1.11; 3.86]	0.869

APACHE II, Acute Physiology and Chronic Health Evaluation II; BDG, (1,3)-β-D-glucan; GIT/HPB, gastrointestinal/hepatobiliary; RRT, renal replacement therapy; SOFA, sequential organ failure assessment; T4/T8, CD4+T/CD8+T ratio.

P-value for the comparison between no IC, and IC groups.

**TABLE 2:** Clinical characteristics of patients in the training cohort at IAI onset according to IAC diagnosis.

Variables	Non IAC (n = 132, 66.7%)	IAC (n = 66, 33.3%)	p-value
Mean age (y)	73 [69; 79]	74 [70; 80]	0.167
Gender, male, n (%)	84 (63.6)	41 (62.1)	0.958
Diabetic mellitus, n (%)	23 (17.4)	13 (19.7)	0.845
Chronic renal failure, n (%)	16 (12.1)	15 (22.7)	0.084
Hepatic falure, n (%)	4 (3.0)	2 (3.0)	0.999
Solid tumor, n (%)	45 (34.1)	19 (28.8)	0.555
Immune system disease, n (%)	1 (0.8)	5 (7.58)	0.016
Hematological disease, n (%)	7 (5.3)	2 (3.0)	0.72
APACHE II score≥15	94 (71.2)	52 (78.8)	0.332
SOFA score	8.00 [5.75; 11.00]	8.00 [5.00; 10.00]	0.672
BDG positivity, n (%)	16 (12.1)	20 (30.3)	0.003
Need for mechanical ventilation, n (%)	128 (97.0)	64 (97.0)	0.999
Need for vasopressor, n (%)	124 (93.9)	61 (92.4)	0.763
Need for RRT, n (%)	16 (12.1)	29 (43.9)	<0.001
High-dose corticosteroids receipt, n (%)	21 (15.9)	16 (24.24)	0.221
Broad-spectrum antibiotics receipt, n (%)	108 (81.8)	59 (89.4)	0.240
Total parenteral nutrition, n (%)	113 (85.6)	61 (92.4)	0.248
Emergency GIT/HPB surgery, n (%)	83 (62.9)	50 (75.8)	0.097
Gastrointestinal perforation, n (%)	52 (39.4)	40 (60.6)	0.008
WBC (cells/mm^3^)	8,000 [5,750; 11,000]	12,370 [8,260; 16,950]	0.178
LY (cells/mm^3^)	602 [416; 905]	695 [500; 979]	0.189
T cells (cells/mm^3^)	440 [256; 667]	503 [353; 736]	0.072
CD4+T (cells/mm^3^)	248 [139; 366]	272 [148; 417]	0.772
CD28^+^CD4+T (cells/mm^3^)	230 [128; 324]	230 [167; 490]	0.881
CD8+T (cells/mm^3^)	120 [75; 205]	145 [99; 213]	0.104
CD28+CD8+T (cells/mm^3^)	57 [35; 94]	34 [23; 54]	<0.001
DR+CD8+T (cells/mm^3^)	54 [32; 87]	84 [45; 128]	0.006
CD38+CD8+T (cells/mm^3^)	55 [32; 87]	52 [49; 71]	<0.001
T4/T8	1.96 [1.22; 2.90]	2.04 [1.31; 3.05]	0.611

APACHE II, Acute Physiology and Chronic Health Evaluation II; BDG, (1,3)-β-D-glucan; GIT/HPB, gastrointestinal/hepatobiliary; RRT, renal replacement therapy; SOFA, sequential organ failure assessment; T4/T8, CD4+T/CD8+T ratio.

p < 0.05, with significant differences for the comparison between no IAC, and IAC groups.

### 3.2 Selection of RF model variables and multivariate logistic regression analysis

**FIGURE 2:**
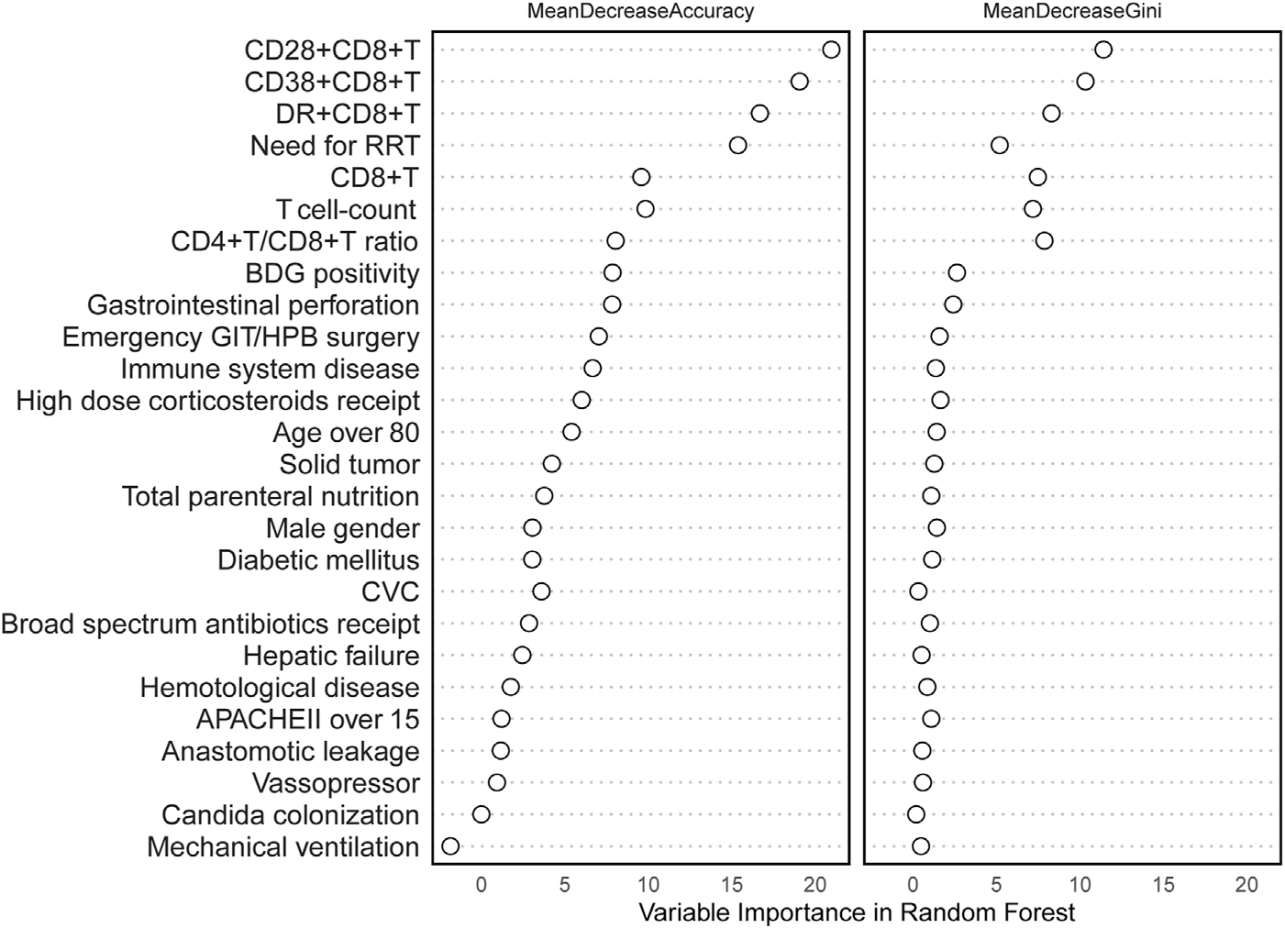
Random forest model variable importance ranking.

All variables were incorporated into the RF model to ascertain the optimal hyperparameter metrics, specifically a metric of 3 and a total number of trees of 500. To determine the significance of each variable, a cross-grid search method was employed (as depicted in Figure 2). Subsequently, the top ten cross-overlapping variables of “MeanDeceAccuracy” and “MeanDecreaseGini” in the importance ranking of RF variables were included in the multivariate logistic backward analysis; the results showed that gastrointestinal perforation, RRT, T-cell count, CD28^+^CD8^+^ T-cell count and CD38+CD8+ T-cell count were independent predictors of IAC in elderly patients (Table 3). The VIF of the variables in the model were found to be within acceptable limits, at 1.188, 1.091, 2.547, 2.499 and 1.430.

**TABLE 3:** Univariable and multivariable logistic regression for predicting IAC in the training cohort.

Risk factors	Univariable analysis			Multivariable analysis		
	OR	95% CI	p-value	OR	95% CI	p-value
CD28+CD8+T	0.99	0.982–0.996	0.005	0.975	0.961–0.985	<0.001
CD38+CD8+T	1.006	1.002–1.009	0.001	1.009	1.004–1.014	0.001
DR+CD8+T	1.003	1–1.007	0.09			
Need for RRT	5.682	2.82–11.83	<0.001	7.058	3.068–17.27	<0.001
CD8+T	1.000	0.998–1.003	0.328			
T cell-count	1.000	0.999–1.001	0.43	1.002	1.000–1.003	0.005
CD4+T/CD8+T ratio	1.060	0.918–1.221	0.414			
BDG positivity	3.152	1.509–6.695	0.002			
Gastrointestinal perforation	2.367	1.3–4.371	0.005	3.826	1.772–8.785	0.001
Emergency GIT/HPB surgery	1.845	0.964–3.664	0.071			

### 3.3 Development and validation of a nomogram based on lymphocyte subsets

**FIGURE 3:**
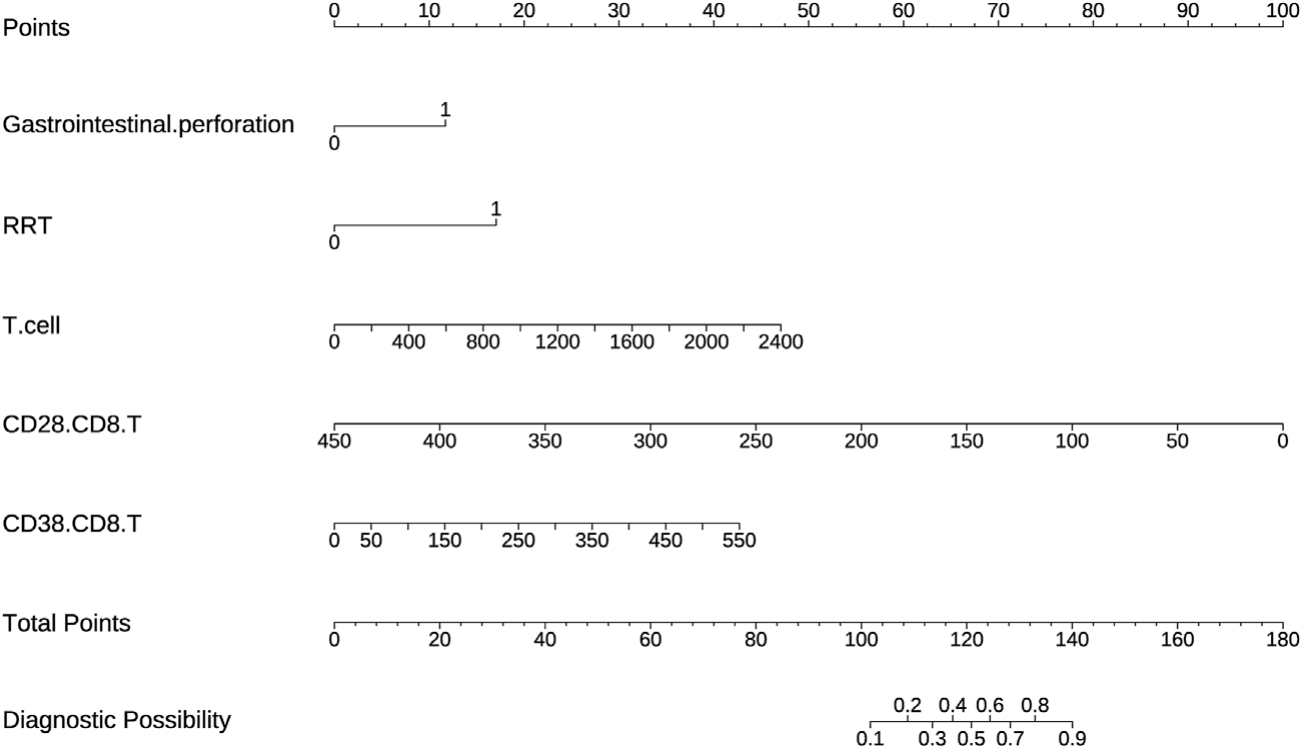
A nomogram based on lymphocyte subsets was used to predict IAC in elderly septic patients.

Statistically significant variables were selected using logistic regression with backward selection to identify risk factors associated with IAC. These independent predictors, including gastrointestinal perforation, RRT, T-cell count, CD28+CD8+ T-cell count and CD38+CD8+ T-cell count, were used to prepare a predictive nomogram using the “rms” package (Figure 3). Each predictor variable corresponded to a distinct score along the horizontal axis of the nomogram. The scores associated with each predictor variable were then aggregated to compute an overall score. By referencing the total score corresponding to the predicted risk of IAC at the base of the nomogram, it was evident that patients exhibiting higher aggregate scores were more susceptible to developing IAC. For example, for a patient who had gastrointestinal perforation, who had a need for RRT, who had a T cell-count of 1,600, a CD28+CD8+T cell-count of 100, a CD38+CD8+T cell-count of 200, the aggregate score was 154, indicating that the IAC risk exceeded 0.9.

**FIGURE 4:**
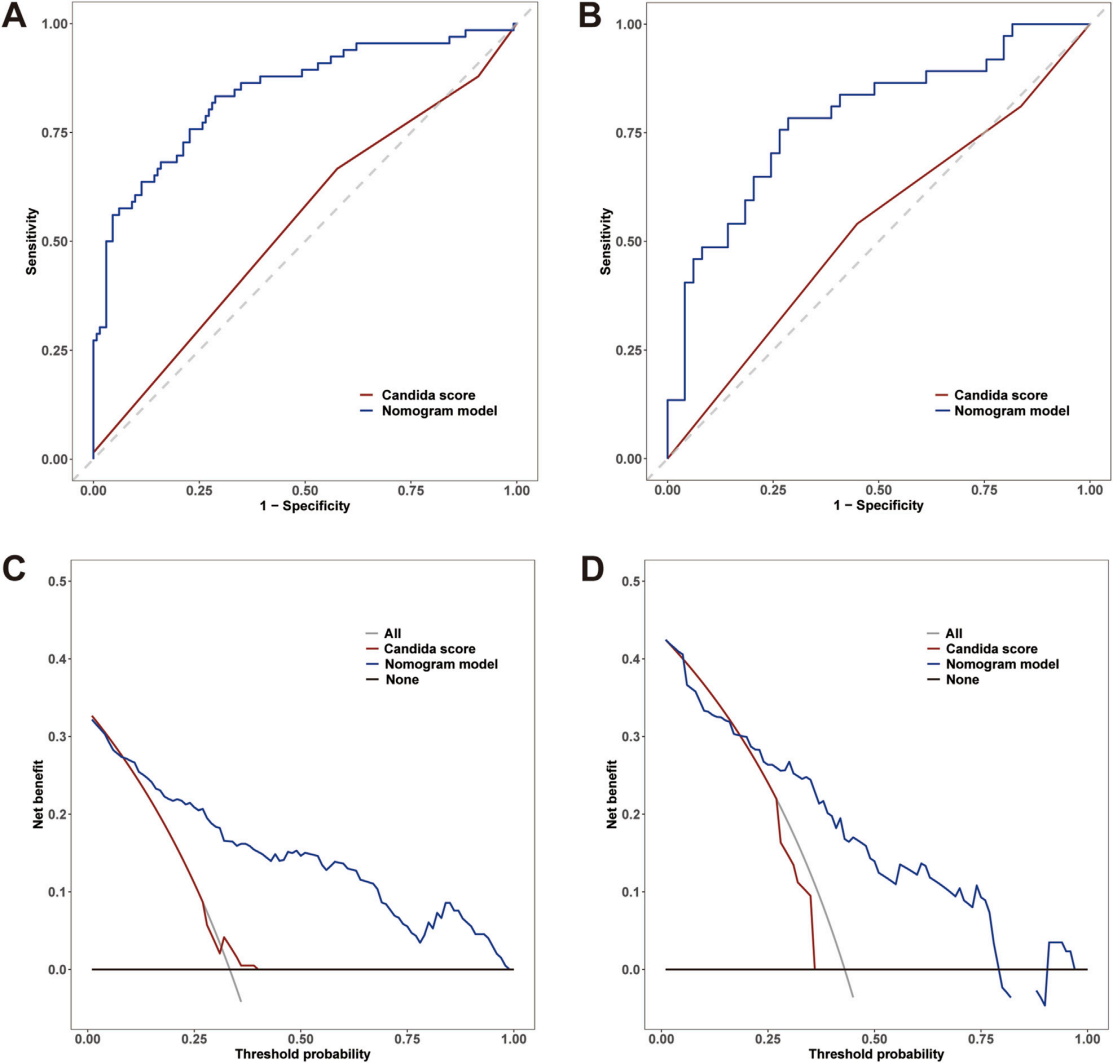
**(A)** Receiver operating characteristic (ROC) curves of the nomogram and *Candida* scores for predicting IAC in elderly septic patients in the training cohort; **(B)** ROC curves in the testing cohort. **(C)** Decision curve analysis (DCA) for the nomogram and *Candida* score in the training cohort; **(D)** DCA curves in the testing cohort.

**FIGURE 5:**
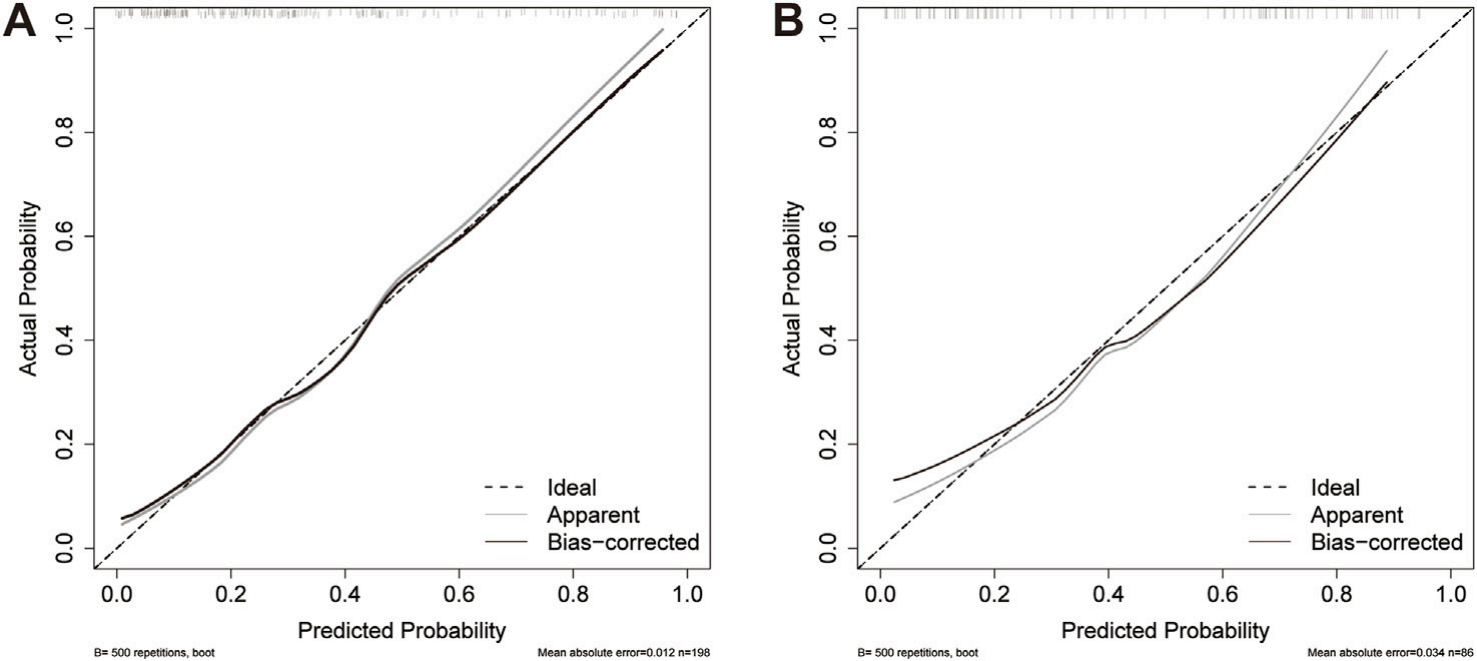
Calibration curves for the nomogram. **(A)** Training cohort; **(B)** Testing cohort. The dashed line indicates the reference line where an ideal nomogram would be. The solid grey lines indicate the performance of the nomogram, while the solid black lines indicate bias correction in the nomogram.

The AUC values of the nomogram in the training and testing cohorts were 0.840 (95% CI 0.778-0.902) and 0.783 (95% CI 0.682-0.883), respectively (Figure 4), indicating that the model had good discrimination. In the training cohort, the Brier scores were 0.141 and the calibration slope was 1.000, indicating that the model had good predictive calibration. The calibration curves for both the training and the test cohorts exhibited strong concordance between the predicted and observed values (Figure 5). The Hosmer-Lemeshow chi-square tests revealed nonsignificant values of 5.876 (*p* = 0.661) and 11.331 (*p* = 0.184) for the respective cohorts, further confirming the model’s predictive accuracy. Additionally, DCA demonstrated that the threshold probabilities for the prediction model range from 0.08 to 0.98 in the training cohort and from 0.18 to 0.80 in the test cohort (Figure 4). Across this wide and clinically relevant range of threshold probabilities, the nomogram exhibited superior overall net benefit, indicating its potential for significant clinical utility.

### 3.4 Clinical utility of the nomogram model

**FIGURE 6:**
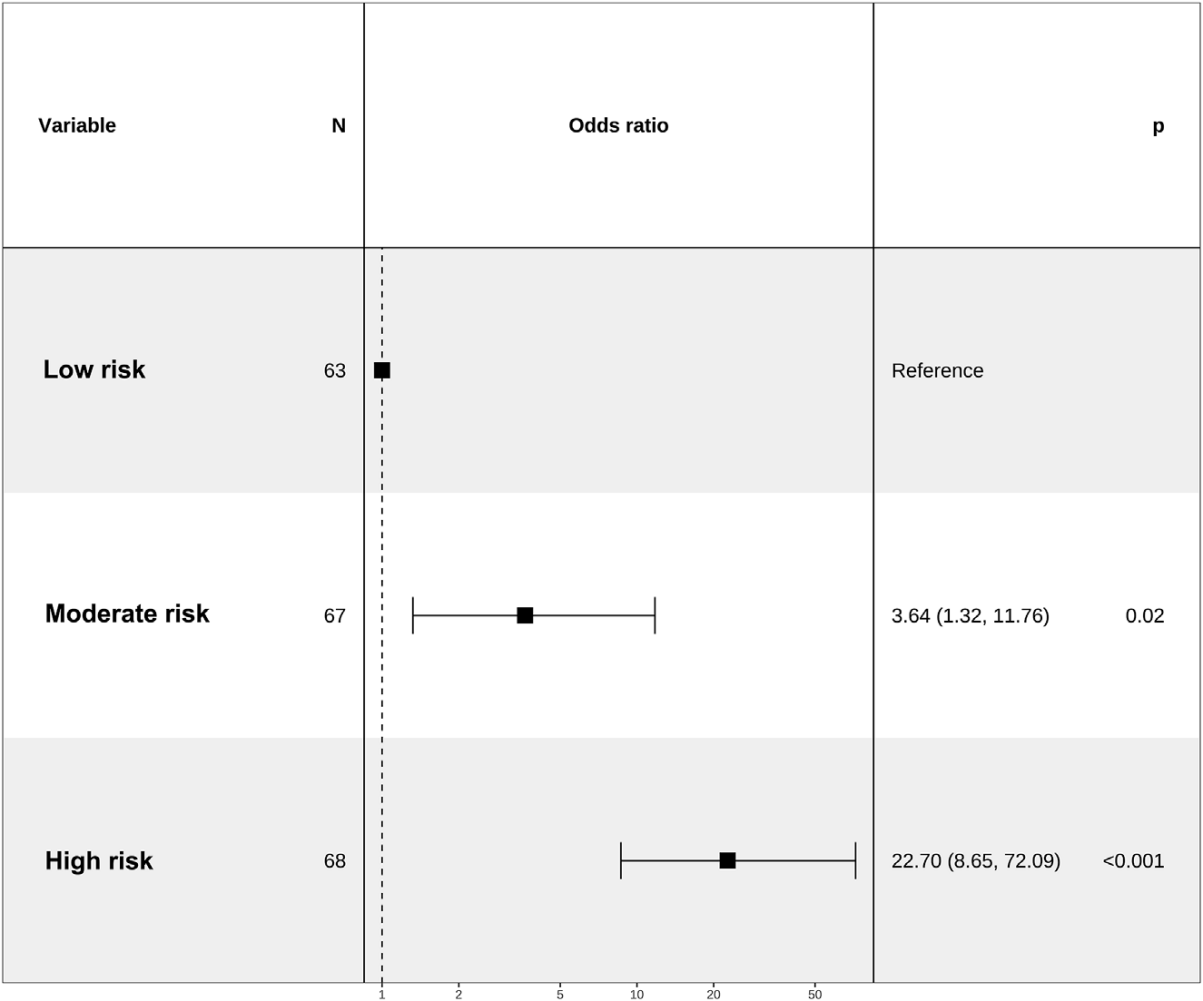
Association between the total nomogram score and IAC risk.

We categorized the nomogram scores into three distinct groups, namely, the low-risk (ranging from 67 to 106 points), the moderate-risk group (spanning from 106 to 116 points), and the high-risk group (encompassing scores from 116 to 159 points). This stratification was undertaken to enhance the clinical utility of the risk score. Notably, the risk of IAC increased in tandem with the overall score. Patients in the moderate-risk and high-risk group exhibited a significantly greater IAC risk than did those in the low-risk group (Figure 6).

### 3.5 Comparison of the nomogram model with the *Candida* score

In the training cohort, a comparative analysis was conducted between the nomogram model and the *Candida* score. The results indicated that the nomogram model exhibited a greater AUC for predicting IAC than did the *Candida* score [0.840 (95% CI 0.778-0.902) vs. 0.539 (95% CI 0.464-0.615), p< 0.001] (Figure 4A). The AUC of the nomogram model in the testing cohort was also greater than that of the *Candida* score [0.783 (95% CI 0.682-0.883) vs. 0.531 (95% CI 0.416-0.647), p< 0.001] (Figure 4B). Those results indicated that the nomogram model had better discriminative ability. Compared with those of the *Candida* score, the nomogram NRIs of the training cohort and testing cohort were 0.5 (95% CI 0.374-0.627, p< 0.001) and 0.546 (95% CI 0.367-0.726, p< 0.001), respectively; the IDIs were 0.350 (95% CI 0.273-0.427, p< 0.001) and 0.369 (95% CI 0.265-0.473, p< 0.001), respectively. The DCA also demonstrated that the nomogram model had a greater net clinical benefit than the superior discriminatory power compared to the *Candida* score (Figures 4C, D).

### 3.6 Nomogram model established by independent predictors plus BDG positivity

**FIGURE 7:**
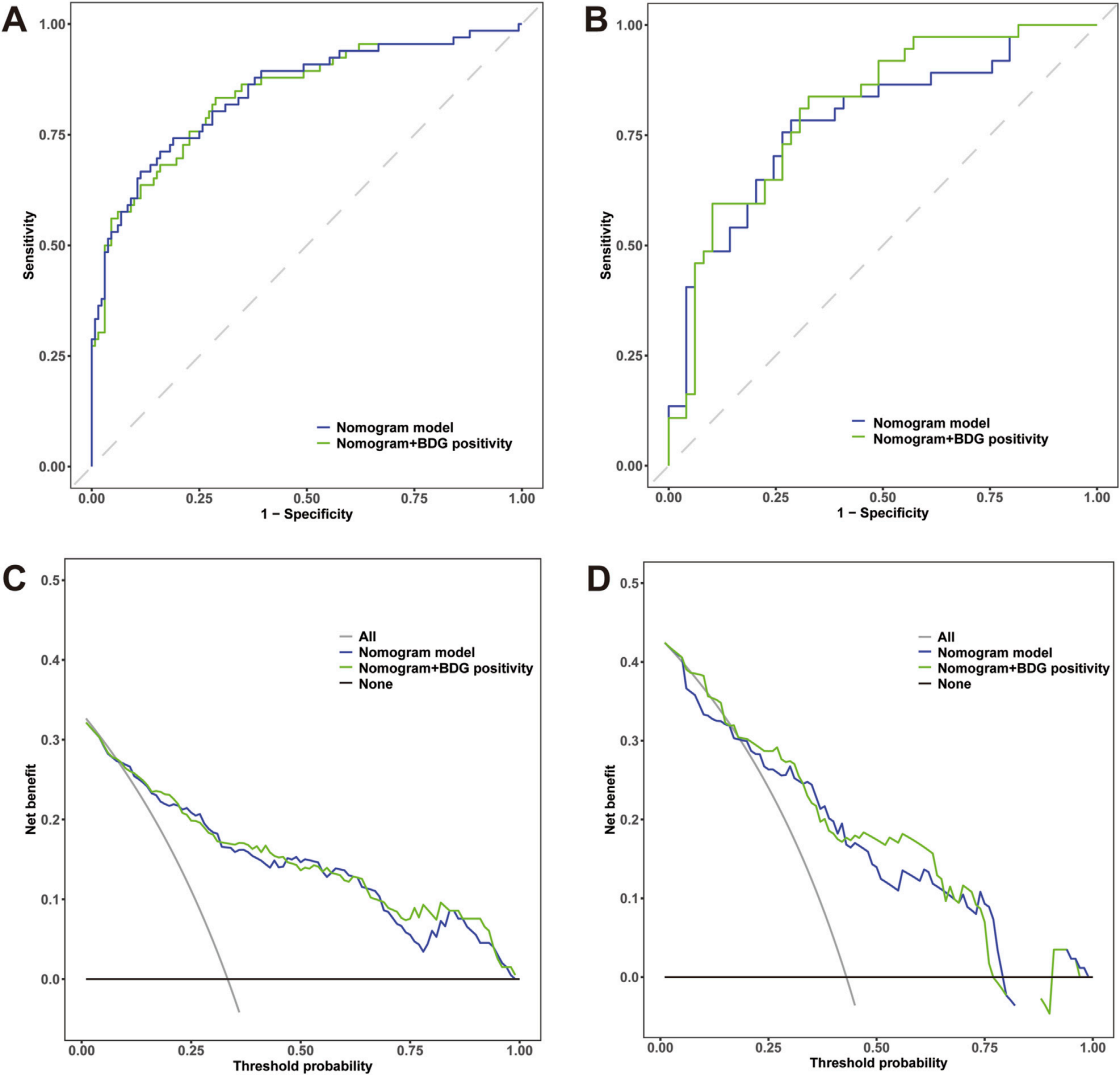
**(A)** Receiver operating characteristic (ROC) curves of the nomogram with independent predictors alone and plus BDG positivity for predicting IAC in elderly septic patients in the training cohort; **(B)** ROC curves in the testing cohort. **(C)** Decision curve analysis (DCA) for the nomogram with independent predictors alone and plus BDG positivity in the training cohort; **(D)** DCA curves in the testing cohort.

BDG has been shown to be useful in identifying IAC in critically ill patients. Although the significant effect of BDG positivity on IAC diagnosis was not observed in the multivariate analysis, we established a new nomogram by independent predictors plus BDG positivity on the basis of clinical validity. ROC analysis was generated to assess the discrimination abilities of two nomograms. Although no statistical significance was observed, the nomogram constructed by independent predictors plus BDG positivity had higher AUC value in predicting IAC in the training cohort [0.845 (95% CI 0.783-0.906) vs. 0.840 (95% CI 0.778-0.902), *p* = 0.561] (Figure 7A) and in the testing cohort [0.809 (95% CI 0.717-0.9) vs. 0.783 (95% CI 0.682-0.883), *p* = 0.122] (Figure 7B). We performed DCA to evaluate the clinical utility and net clinical benefits that two nomograms would bring to patients and the results revealed that the integrated nomogram with BDG positivity was superior to the nomogram with independent predictors only (Figures 7C, D).

## 4 Discussion

Based on our current understanding, this study represents the initial effort to elucidate the risk factors associated with IAC among elderly septic patients. In this study, we demonstrated that new biomarkers, T cell-count, CD28+CD8+T cell-count and CD38+CD8+T cell-count, were significantly altered in elderly septic patients with IAC. Moreover, we developed a nomogram for the early diagnosis of IAC in elderly septic patients. The nomogram, based on gastrointestinal perforation, need for RRT, T cell-count, CD28+CD8+T cell-count and CD38+CD8+T cell-count, had a good identifiable value for IAC, with an AUC of 0.840.

Over the past decade, a substantial accumulation of evidence has been documented, suggesting the involvement of *Candida* species as a significant contributor to the pathogenesis of IAI (Bassetti et al., 2022). Presently, *Candida* is widely acknowledged as a leading causative agent of IAI, owing to its strong correlation with significant morbidity, mortality, and escalated healthcare expenditures (Klingspor et al., 2015). Furthermore, any delays in initiating antifungal therapy have the potential to exacerbate mortality rates. In contrast, the excessive prescription of antifungal agents poses significant risks, including exposure to drug toxicity and the promotion of resistance (Azoulay et al., 2012). Prompt identification of IAC is paramount. However, this approach is constrained by nonspecific clinical manifestations, prolonged waiting times for culture results, and logistical challenges encountered during sampling of potential infection sites within deep tissue. Despite the advent of novel diagnostic biomarkers, such as BDG, their widespread accessibility remains to be established, and they are prone to false-positive outcomes. Given these considerations, there is a pressing need for strategies that aim to provide timely antifungal therapy to elderly septic patients who are at a heightened risk of developing IAC.

Multiple risk models have been documented for forecasting IC among patients admitted to the ICU. However, a significant proportion of these investigations rely heavily on retrospective data analysis (Playford et al., 2016), or they narrowly focus on candidaemia, despite the fact that IAC also comprises a substantial segment of IC. Unfortunately, our understanding of the risk factors associated with IAC has predominantly stemmed from studies encompassing patients with candidaemia or those experiencing nonpostoperative IAI (Dupont et al., 2015; Xia and Wang, 2019). The aforementioned studies predominantly concentrated on community-acquired infections, displaying unique features that may not be directly applicable to septic patients in the ICU, who are inherently predisposed to IAC. In contrast, the current study provides a more precise depiction of routine clinical practice, as it exclusively includes elderly septic patients admitted to the ICU, reflecting the significant proportion of patients afflicted by IAC.

The current study has identified various clinical risk factors, all of which have been previously implicated in the diagnosis of IAC (Bassetti et al., 2022). Gastrointestinal perforation results in the disruption of the gastrointestinal barrier, thereby establishing a conducive environment that encourages the proliferation of *Candida* cells within the peritoneal cavity. Additionally, breaches in mucosal barriers increase the likelihood of translocation of the *Candida* species into the bloodstream (Pemán et al., 2017). The impact of need for RRT on risk of IAC is likely multifactorial. There is evidence of innate and adaptive immune dysregulation in the setting of renal failure. Moreover, patients those with haemodialysis catheters are also at elevated risk of IAC owing to the loss of integrity of the normal anatomical defences (Lass-Florl et al., 2024).

The present study is believed to be the first prospective study to use lymphocyte subtyping assessing the quantitative changes in host immune status and the potential role of these immune parameters in the diagnosis of IAC in elderly septic patients. We found a significant correlation between T-cell count, CD28+CD8+ T-cell count and CD38^+^CD8^+^ T-cell count and the presence of IAC in elderly septic patients. As is known to all, the ability of the host to survive IAC requires a well-coordinated response by the innate and adaptive immune systems; both of which are often impaired in patients with invasive candidiasis (Zhang et al., 2019). Most patients with IAC are immunosuppressed due to aging and underlying disease. Although CD4+T cells have a central role in coordination of the host defenses against fungi, the cytotoxic effect of CD8+T-cells seems ultimately to be essential for termination of the infection. We have previously demonstrated that CD8+T immunity is impaired in response to invasive candidiasis through the mammalian target of rapamycin signaling pathway (Zhang et al., 2021). T-cell activation is carefully regulated by expression of positive and negative co-stimulatory molecules that prevent unbridled T-cell function. We detected significant alterations in the T cell compartment in elderly IAC patients. CD28 acts as a classic co-stimulating molecule on the surface of T cells, implicates in a wide array of T-cell responses, including T-cell proliferation, antigen-stimulated differentiation, activation and cytokine production. Our previous study identified CD28+CD8+T cell-count as an independent risk factors for invasive candidiasis and early mortality in critically ill patients (Zhang et al., 2019), which is consistent with the present study. CD38 is a surface glycoprotein existing on many immune cells. As a marker of cell activation, it is associated with many infectious diseases. In certain infections, CD38+CD8+T cells undergo a rapid increase after the infection once the immune control of acute phase is achieved (Cao et al., 2011; Piedra-Quintero et al., 2020). However, persistency of immune activation and CD38^+^ expression may reflect the failure of the host’s immune system to fully suppress pathogens. We demonstrated that CD38+CD8+T cells were more prominent in IAC patients. It is possible that CD38+CD8+T cells represent overactivated, potentially exhausted, and less efficient T cells in *Candida* control, as suggested by SARS-CoV-2+ and HIV studies (Rebillard et al., 2021). These results of the present study provide evidence that T-cell count, CD28+CD8+ T-cell count and CD38+CD8+ T-cell count, which are quickly available through lymphocyte subtyping, are important for the early diagnosis of IAC in elderly septic patients in clinical practice, facilitating timely intervention and improving patient outcomes.

Although numerous reports have established a notable correlation between all five risk factors and the diagnosis of IAC, current research indicates that none of these factors alone serves as a definitive indicator for identifying patients with a high risk of developing IAC, thus justifying the commencement of antifungal therapy (Bassetti et al., 2022; Lass-Florl et al., 2024). Given the accessibility and swiftness in acquiring data on all five factors, the formulation of a predictive nomogram that incorporates these factors, encompassing clinical risk factors and lymphocyte subsets, emerges as a promising strategy for the early detection of IAC. This nomogram demonstrated excellent discriminative ability, with an AUC of 0.840 in the training cohort, and its validity was further confirmed in the testing cohort (AUC = 0.783). Furthermore, the *Candida* score is extensively employed for diagnosing IC in patients admitted to the ICU (León et al., 2009). However, its reliance on fungal culture results, which typically require at least 2 days to obtain, does not expedite the diagnostic timeline. Our current research underscores the *Candida* score’s limited diagnostic efficacy in the context of IAC in the elderly septic patients. Conversely, our proposed nomogram exhibits superior discriminatory capability compared to the *Candida* score. This finding suggests that the nomogram offers enhanced diagnostic precision for the early detection of IAC. Additionally, the positive net benefits demonstrated by DCA within the predictive model indicate its promising clinical utilization.

The nomogram was a reliable tool for clinical decision-makers to predict the risk of IAC in elderly septic patients. Based on the nomogram scores, we have delineated three distinct risk groups. The forest plot analysis reveals that the nomogram possesses remarkable predictive prowess and significant potential for clinical utilization. In clinical settings, patients belonging to the low-risk category, scoring between 67 and 106 points, exhibit a conspicuously low incidence of IAC and do not require antifungal therapy. Conversely, the high-risk group, scoring between 116 and 159 points, demonstrates a significantly elevated incidence of IAC and may benefit from the initiation of antifungal agents. Patients classified as moderate risk, scoring between 106 and 116 points, may require further assessment, and the necessity for antifungal therapy remains indeterminate. The utilization of advanced diagnostic modalities, including next-generation sequencing, has the potential to provide valuable insights for this patient cohort. Notably, the nomogram score exhibits a robust positive correlation with the actual risk of IAC. The parameters encompassed within the nomogram are readily accessible, economically feasible, and reliable in clinical practice. Therefore, the nomogram can be widely utilized and serves as a practical tool for physicians to assess the risk of IAC among septic patients at the bedside. Further research is crucial to validate the efficacy of the nomogram in guiding the initiation or discontinuation of antifungal therapy. Since BDG has been shown to be useful in identifying IAC in critically ill patients, we established a new nomogram by independent predictors plus BDG positivity on the basis of clinical validity. Although no statistical significance was observed, the nomogram constructed by independent predictors plus BDG positivity had higher AUC value in predicting IAC. This result demonstrated the potential role of BDG in diagnosing IAC. In future researches, fungal biomarkers such as BDG and *Candida* PCR should be considered for optimization of the predicting model.

The strength of this study is the concentration on elderly septic patients in the ICU, as this demographic group is especially susceptible to infections due to their weakened immune system. *Candida* spp. are typically the resident microflora that colonize the gastrointestinal tracts. However, as individuals get older, metabolic disorders can weaken the mucosal barriers, allowing these colonized *Candida* strains to penetrate and invade deeper into the body’s organs (Gong et al., 2020). To the best of our knowledge, this study is the first to highlight the characteristics of IAC in the elderly septic population. However, it is worth noting that there are indeed several limitations in this study. Firstly, it is noteworthy that the data were procured specifically from one hospital located in China, and the study was confined to ICU patients only. Because the study is a single-center cohort, our study is limited in its generalizability due to selection bias. Consequently, the extrapolation of the results to the broader elderly population should be approached with caution. Despite this inherent limitation, our validation efforts have yielded remarkably satisfactory AUC values and calibration metrics in both the training and testing cohorts, surpassing the *Candida* score in terms of its discriminatory capabilities. Secondly, there exists the potential risk of misclassification between intra-abdominal candidiasis (IAC) and non-IAC due to the sensitivity of peritoneal culture. So rigorous adherence to a standardized protocol has been implemented. This protocol emphasizes the direct inoculation of peritoneal samples into an optimized culture medium, with cultures being stored for a maximum of 14 days to facilitate detection of delayed positivity. By following this protocol, the aforementioned risk of misclassification is mitigated to a considerable degree. Thirdly, although the nomogram presented in our study effectively facilitates early and precise diagnosis of IAC among elderly septic patients, it is crucial to acknowledge that our analysis was inherently limited in terms of controlling for certain variables. Notably, we were unable to account for specific factors such as the type of abdominal surgery undergone by the patients or the exact site of perforation or leakage. These variables have been extensively documented in previous research to have a substantial impact on the frequency of *Candida* isolation from abdominal fluid samples. Fourthly, we focused on a single snapshot of lymphocyte subtyping at the onset of IAI to formulate a model for the rapid diagnosis of IAC. What we should note is that static measurement of immune markers can fail to capture the nuances of immunological progression in critically ill patients. Future research should conduct a time-series analysis of lymphocyte subtyping to better capture the trajectory of the immune response in IAC. Given the preliminary nature of our findings, it is crucial to acknowledge that the clinical utility of our nomogram, while promising, necessitates further validation. To comprehensively assess its clinical performance, we advocate for the conduct of larger-scale, multi-center, prospective studies applying this model to a diverse patient cohort. Such endeavors will not only solidify the evidence base but also justify the allocation of resources towards more exhaustive longitudinal research endeavors. It is imperative to view our current results as a stepping stone towards a more definitive understanding of the nomogram’s potential to inform clinical decision-making.

## 5 Conclusion

To summarize, our findings indicate a significant correlation between T-cell count, CD28+CD8+ T-cell count and CD38+CD8+ T-cell count and the presence of IAC in elderly septic patients. By creating a predictive nomogram incorporating factors such as gastrointestinal perforation, RRT and the lymphocyte subtyping markers, clinicians can promptly assess an elderly patient’s risk of developing IAC early on the infection process. This tool holds the potential to enhance the prognosis for elderly septic patients with IAC by facilitating timely intervention and management strategies.

## Data Availability

The raw data supporting the conclusions of this article will be made available by the authors, without undue reservation.
